# Synthetic autotrophic yeast enables high itaconic acid production from CO_2_*via* integrated pathway and process design

**DOI:** 10.1039/d5gc03149d

**Published:** 2025-09-24

**Authors:** Özge Ata, Lisa Lutz, Michael Baumschabl, Diethard Mattanovich

**Affiliations:** a BOKU University, Vienna, Institute of Microbiology and Microbial Biotechnology, Department of Biotechnology and Food Science 1190 Vienna Austria oezge.ata@boku.ac.at; b Austrian Centre of Industrial Biotechnology Vienna 1190 Austria

## Abstract

Single carbon (C1) substrates are gaining importance as future feedstocks for the production of bio-based chemicals. Carbon dioxide, a major greenhouse gas, offers a promising alternative to the traditional feedstocks to shift towards C1-based, sustainable processes. Here, we present a synthetic autotrophic *Komagataella phaffii* (*Pichia pastoris*) that is able to produce itaconic acid by the direct conversion of CO_2_, achieving final titers of approximately 12 g L^−1^ in bioreactor cultivations. We show that a combined approach that integrates balancing the flux between the Calvin–Benson–Bassham (CBB) cycle and itaconic acid metabolism with process design was essential to enhance the production. Our study demonstrates the potential of *K. phaffii* as a microbial platform using CO_2_ as the direct carbon source, aligning with the future goals of establishing sustainable bioprocesses.

## Abstract 2

We show that combining pathway and process engineering enables synthetic autotrophic *K. phaffii* to produce ∼12 g L^−1^ itaconic acid from CO_2_, highlighting the role of interplay between native and heterologous metabolic pathways and process design.

## Graphical abstract

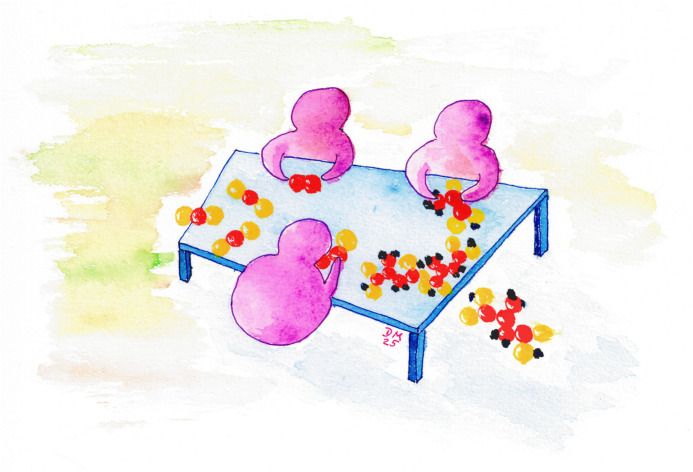

**Green foundation**

1. Our work advances green chemistry by establishing a platform that converts CO_2_—a major greenhouse gas—into itaconic acid, a valuable bio-based chemical. By engineering *Komagataella phaffii* to operate autotrophically using a synthetic Calvin– Benson–Bassham (CBB) cycle, we eliminate reliance on sugar-based or fossil-derived feedstocks.

2. We achieved titers of ∼12 g L^−1^ itaconic acid directly from CO_2_, demonstrating a scalable alternative to conventional production using sugar or fossil-based substrates.

3. This process could be further improved by enhancing CO_2_ fixation efficiency, integrating renewable energy inputs, combining with other CO_2_-derived substrates and expanding the product spectrum. Future work may include life cycle assessments to quantify environmental impact and guide optimization toward fully sustainable, CO_2_-negative C1-based bioprocesses.

## Introduction

As we struggle with the challenges of the climate crisis, it is essential to develop innovative strategies that not only mitigate these issues but also offer solutions for long-term sustainability. Carbon dioxide (CO_2_), one of the major greenhouse gases, is increasing in atmospheric concentration due to societal activities, which endangers our entire planet. In this regard, single carbon (C1)-based bioprocesses emerge as a promising technology to reduce CO_2_ emissions, *via* the microbial conversion of CO_2_ or other CO_2_-derived C1-sources such as methanol and formate.^1–3^

**Fig. 1:**
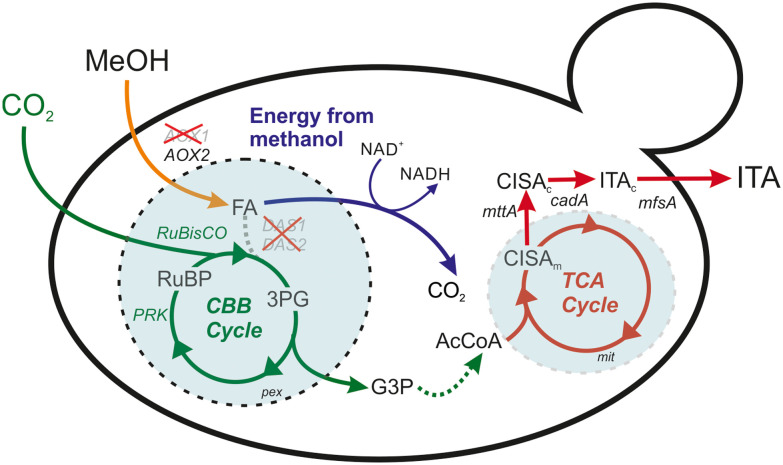
Metabolic pathway of the itaconic acid production in the synthetic autotrophic *K. phaffii.* Deletion of *DAS1* and *DAS2* interrupts methanol assimilation (dashed gray line). *AOX1* was knocked out to reduce the rate of formaldehyde formation which could be toxic to the cells. More details about the engineering strategy can be found in ref. 22. Expression of the key enzyme *cadA* among with two transporters, *mttA* and *mfsA*, enables itaconic acid production from CO_2_. 3PG: 3-phosphoglycerate, AcCoA: acetyl-coenzyme A, *AOX1* and *AOX2*: alcohol oxidase 1 and 2, *cadA*: *cis*-aconitate decarboxylase, CBB cycle: Calvin–Benson–Bassham cycle, CISA_c_: cytosolic *cis*-aconitate, CISA_m_: mitochondrial *cis*-aconitate, *DAS1* and *DAS2*: dihydroxyacetone synthase 1 and 2, FA: formaldehyde, G3P: glyceraldehyde 3-phosphate, ITA: itaconic acid, *mttA*: mitochondrial tricarboxylic acid transporter, NAD^+^/NADH: nicotinamide adenine dinucleotide, *PRK*: phosphoribulokinase, RuBP: ribulose 1,5-bisphosphate, *RuBisCO*: ribulose 1,5-bisphosphate carboxylase/oxygenase. pex: peroxisome, mit: mitochondria.

Several natural or engineered autotrophs^4–8^ and acetogens^9–12^ have been harnessed to convert CO_2_ into a broad range of industrially relevant chemicals such as alcohols and organic acids.^13–17^ Among these organic acids, itaconic acid has gained interest worldwide as being one of the 12 top-value added chemicals by the US Department of Energy. The global market size of production of itaconic acid has reached more than 100 million USD in 2024 and expected to exceed more than 170 million USD by 2031.^18^ Itaconic acid can serve as a building block for several products such as plastics, drug carriers, polymer binding agents, resins, and synthetic fibers.^19^ Conventionally, *Aspergillus terreus* is the native, predominant industrial host for the microbial production of itaconic acid.^20,21^ The biosynthesis pathway of itaconic acid involves the key gene *cis*-aconitate decarboxylase, *cadA*, which converts *cis*-aconitate into itaconic acid (Fig. 1). Two additional transporters, mitochondrial *cis*-aconitate transporter (MttA) and the major facilitator superfamily transporter (MfsA), transport the substrate *cis*-acotinate from the mitochondria to the cytosol, and the product itself from the cytosol to the extracellular environment, respectively. However, the industrial microbial production of itaconic acid by *A. terreus* and *Ustilago maydis* predominantly relies on sugar-based feedstocks, competing with agricultural land use. A C1-substrate based bioprocess could therefore play a key role in enabling more sustainable production.

In a recent study, we demonstrated that a synthetic autotrophic yeast *Komagataella phaffii* (*Pichia pastoris*), can produce itaconic acid up to 2 g L^−1^ titers in shake flask cultivations using CO_2_ and methanol as the sole carbon and energy sources, respectively; through the introduced CBB cycle. However, upscaling proved challenging, as titers in lab-scale bioreactors reached only around 0.5 g L^−1^, underlining the need for rigorous process design to achieve higher production levels.

In the present study, we seek to increase the itaconic acid production performance of the synthetic autotrophic *K. phaffii* from CO_2_ in lab-scale bioreactor cultivations. Through combined efforts of metabolic engineering to balance the flux of the CBB cycle and itaconic acid metabolism with process parameter optimization, we could achieve 11.84 g L^−1^ ± 0.26 of itaconic acid improving the final titer and specific productivity by 22.5-fold and 5.3-fold respectively, compared to the previous bioreactor cultivation.^16^

## Results

### Moderate dissolved oxygen (DO) levels enhance itaconic acid production, while excessively low DO impairs titers

**Fig. 2:**
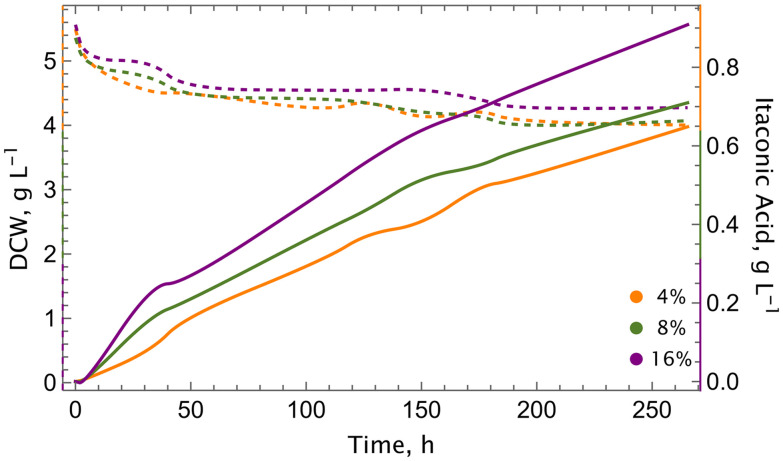
Dissolved oxygen concentration is a key parameter for the production of itaconic acid. Growth (dashed lines) and itaconic acid production (solid lines) profiles of the cadA + mttA strain at different dissolved oxygen concentrations. Experiments were conducted in single runs in lab-scale bioreactors, using 5% CO_2_ in the inlet gas stream, at 30 °C.

Recently, we have demonstrated that the production of itaconic acid is achievable through the conversion of CO_2_ by an engineered autotrophic *K. phaffii* strain.^16^*K. phaffii* is not able to produce itaconic acid as it does not possess a *cadA* gene. Itaconic acid production was enabled by the expression and balancing of *cadA* and *mttA*, resulting in the cadA + mttA strain with a final titer of approximately 0.80 g L^−1^ in shake flasks. Furthermore, it was demonstrated that dissolved oxygen (DO) concentration is a crucial parameter in laboratory-scale fermentations and shown that at high DO concentration (20%), itaconic acid production is lower. Therefore, in the present study, we investigated the setpoints 4%, 8%, and 16% (Fig. 2 and Fig. S3). The growth profile exhibited a uniform pattern across all conditions in bioreactors, with no observable growth, despite the absence of any growth impairments in the tested strain during the shake flask cultivations. However, an obvious trend was observed in the itaconic acid production profiles across varying DO concentrations. The highest titers (0.91 g L^−1^) were observed at 16% O_2_, while the lowest was recorded at 4% (0.65 g L^−1^). Furthermore, analysis of the expression levels of key genes involved in the CBB cycle and itaconic acid metabolism revealed no significant trend in response to varying DO values (Fig. S1). Consequently, 16% DO was selected for subsequent bioreactor experiments, as this condition yielded the highest itaconic acid titers and productivity (Table 1).

**Table 1:** Process parameters in shake flask and bioreactor cultivations during the autotrophic production phase. Unless indicated in parentheses, final titers of Day 8 of the cultivation are given. Dissolved oxygen concentration is not controlled during shake flask cultivations. MC: multicopy

Strain	Cultivation	Parameters				Final titer (g L^−1^)	Final DCW (g L^−1^)	Specific productivity, *q*_*p*_ (mg g DCW^−1^ h^−1^)	Growth rate, *μ* (h^−1^)	Product yield, *Y*^*a*^_*P*/*X*_ or *Y*^*b*^_*P*/*X*_ (g g DCW^−1^)
		Temperature (°C)	Dissolved oxygen (%)	CO_2_ feed (%)	Initial OD					
cadA	Shake flask	25–28	—	10	4	0.304 ± 0.016 (Day 7)	3.03 ± 0.10	1.14 ± 0.030	0.007 ± 0.0001	0.154 ± 0.002
cadA + mttA	Shake flask	25–28	—	10	4	0.628 ± 0.001 (Day 7)	2.31 ± 0.02	3.75 ± 0.000	0.006 ± 0.0000	0.480 ± 0.004
cadA + mttA	Shake flask	25–28	—	10	10	1.433 ± 0.019 (Day 7)	4.39 ± 0.00	2.96 ± 0.022	0.004 ± 0.0001	0.735 ± 0.026
cadA + mttA	Shake flask	25–28	—	10	20	1.944 ± 0.052 (Day 7)	6.33 ± 0.16	2.45 ± 0.033	0.002 ± 0.0002	1.106 ± 0.075
cadA + mttA	Shake flask	25–28	—	5	4	0.766 ± 0.039	2.57 ± 0.16	2.55 ± 0.098	0.006 ± 0.0001	0.458 ± 0.008
cadA + mttA + mfsA_GAP_	Shake flask	25–28	—	5	4	0.913 ± 0.026	2.48 ± 0.09	3.07 ± 0.108	0.005 ± 0.0003	0.577 ± 0.048
cadA + mttA + mfsA_FDH1_	Shake flask	25–28	—	5	4	0.985 ± 0.062	2.39 ± 0.16	3.42 ± 0.197	0.005 ± 0.0005	0.662 ± 0.086
cadA + mttA + mfsA_GAP_	Shake flask	25	—	5	4	1.111 (1.571, Day 11)	3.65 (3.78, Day 11)	3.09	0.008	0.376
MC-CBB_cadA + mttA + mfsA_GAP_	Shake flask	25	—	5	4	1.369 ± 0.020 (2.021 ± 0.026, Day 11)	3.91 ± 0.07 (4.14 ± 0.10, Day 11)	4.15 ± 0.061	0.009 ± 0.0003–0.008	0.463 ± 0.007
MC-CBB-IA_ cadA + mttA + mfsA_GAP_	Shake flask	25	—	5	4	1.908 ± 0.303	3.49 ± 0.32	5.11 ± 0.795	0.008 ± 0.0006	0.657 ± 0.088
cadA + mttA	Bioreactor, YNB + glycerol batch	30	4	5	20	0.649	4.00	0.51	No growth	0.137
cadA + mttA	Bioreactor, YNB + glycerol batch	30	8	5	20	0.710	4.07	0.5	No growth	0.151
cadA + mttA	Bioreactor, YNB + glycerol batch	30	16	5	20	0.908	4.27	0.70	No growth	0.185
cadA + mttA	Bioreactor, YNB + glycerol batch	30	16	10	20	0.648	2.91	0.83	No growth	0.157
cadA + mttA	Bioreactor, YNB + glycerol batch	25	16	10	20	1.389	4.42	1.32	0.0004	0.251
cadA + mttA + mfsA_GAP_	Bioreactor, YNB + glycerol batch	25	16	10	20	1.078	5.32	1.59	0.0007	0.566
cadA + mttA + mfsA_GAP_	Bioreactor, YNB + glycerol batch	30	16	10	20	2.692	2.91	0.90	No growth	0.321
MC-CBB-IA_ cadA + mttA + mfsA_GAP_	Bioreactor, YNB + glycerol batch	25	16	10	20	6.738	8.44	3.22	0.002	1.738
MC-CBB-IA_ cadA + mttA + mfsA_GAP_	Bioreactor, YPG batch	25	16	10	20	11.836 ± 0.262	15.73 ± 0.64	3.94 ± 0.006	0.004 ± 0.0002	0.997 ± 0.037
MC-CBB_cadA + mttA + mfsA_GAP_	Bioreactor, YPG batch	25	16	10	20	5.141 ± 0.057	13.22 ± 0.64	1.93 ± 0.072	0.003 ± 0.0001	0.563 ± 0.040

### Lower temperature and higher CO_2_ concentration lead to higher itaconic acid production

**Fig. 3:**
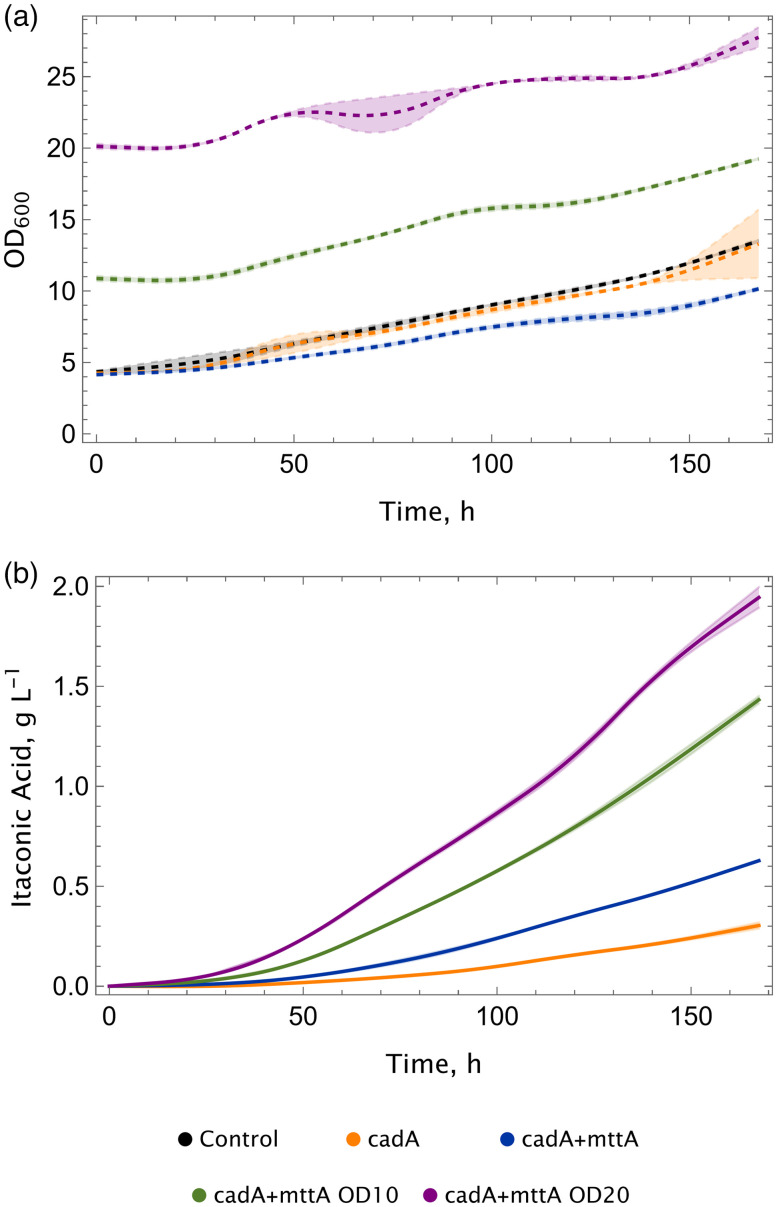
Decreasing temperature improves itaconic acid production. (a) Growth and (b) itaconic acid production profiles of the cadA + mttA strain. Experiments were conducted in duplicates, CO_2_ shakers supplied with 10% CO_2_. The temperature of the shaker was kept between 25–28 °C during the cultivation. Standard deviations (±) are calculated and shown in shades.

Standard shake flask experiments have hitherto been conducted at 30 °C (with 5% CO_2_), as this is the optimum growth temperature of *K. phaffii* on glucose or methanol. In addition, it has been established that the enzymatic activity of CadA increases with elevated temperatures.^23,24^ However, it is important to note that CO_2_ is a gaseous substrate, and its solubility in liquids is higher at lower temperatures. Furthermore, the specificity of RuBisCO towards CO_2_ increases when temperature is decreased.^25^ On the other hand, as we demonstrated in our previous study, elevated CO_2_ concentrations boost the itaconic acid production.^16^ Consequently, the performance of the cadA + mttA strain was examined at 25 °C and compared to 30 °C in shake flask experiments at 10% CO_2_ (Fig. 3).

A 5 °C decrease in temperature resulted in a two-fold impact: Firstly, the growth of the control strain and the production strains was enhanced in comparison with 30 °C. In our previous study, cultivating cells with higher initial ODs did not result in growth, and the ODs remained almost unchanged during the cultivation process. Conversely, at a lower temperature, a growth rate of 0.002 h^−1^ was observed at initial OD = 20 at 25 °C, albeit still lower than the growth rate with initial OD = 4. Furthermore, at an initial OD of 4, the growth rate was found to be 0.006 h^−1^, which is 1.5-fold higher than that observed at 30 °C. Secondly, the final titers of the itaconic acid obtained was enhanced and reached to approximately 1.95 g L^−1^ (compared to 1.71 g L^−1^ at 30 °C with 10% CO_2_).^16^

**Fig. 4:**
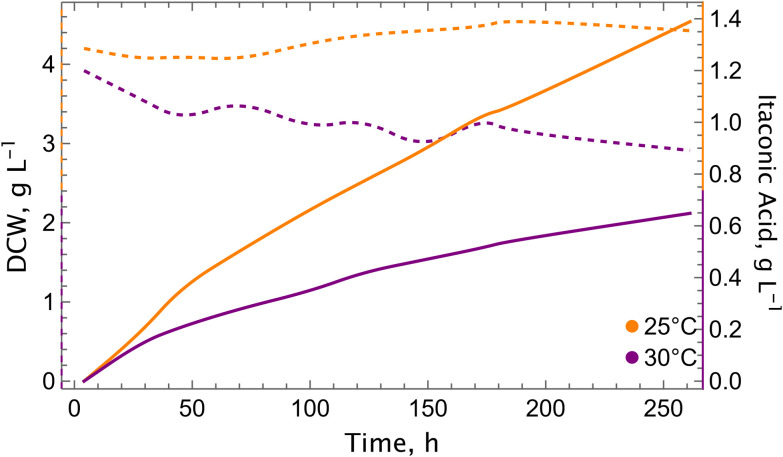
Cultivation at a lower temperature increases itaconic acid production in the bioreactor. Growth (dashed lines) and itaconic acid production (solid lines) profiles at 25 °C (green) and 30 °C (orange) with the cadA + mttA strain. Experiments were conducted in single runs in lab-scale bioreactors, using 10% CO_2_ in the inlet gas.

Due to technical limitations during shake flask cultivations, it was not possible to maintain a constant temperature of 25 °C, which fluctuated between 25 and 28 °C. Consequently, we wanted to assess the impact of temperature in a more controlled environment, and conducted bioreactor cultivations. As anticipated, the results demonstrated the positive impact of reduced temperatures on both growth and itaconic acid production performance (Fig. 4). The growth was close to zero (*μ* = 0.0004 h^−1^) for the cultivations at 25 °C, whereas a decrease in the biomass was observed during the cultivation at 30 °C. The final titers and specific productivity were increased 2.1-fold and 1.6-fold, respectively, compared to 30 °C (Table 1). The final itaconic acid titer was 1.39 g L^−1^ (*versus* 0.65 g L^−1^ at 30 °C), which is the highest concentration recorded among the bioreactor experiments conducted so far. Furthermore, the expression levels of key genes involved in the CBB cycle and itaconic acid metabolism were analysed (see Fig. S2). We took samples at three different time points. The expression levels of the measured genes from the CBB cycle (*PRK*, RuBisCO), the TCA cycle (*CIT1*, *ACO1*, *ACO2*), and itaconic acid metabolism (*cadA*, *mttA*) were found to be elevated at 25 °C in comparison to 30 °C during the 148 hours of bioreactor cultivation, with the exception of the final time point (194 hours of the cultivation).

### Co-expression of *mfsA* leads to improvement of itaconic acid titers

Following the identification of the key process parameters, we shifted our focus to metabolic engineering to improve the strain efficiency. We introduced the *mfsA* gene to the cad + mttA strain to test whether co-expression would increase the itaconic acid production. Utilising two strong promoters, pFDH1 (methanol inducible) and pGAP (constitutive), the impact of *mfsA* on itaconic acid production in the cadA + mfsA strain was examined in shake flasks.

**Fig. 5:**
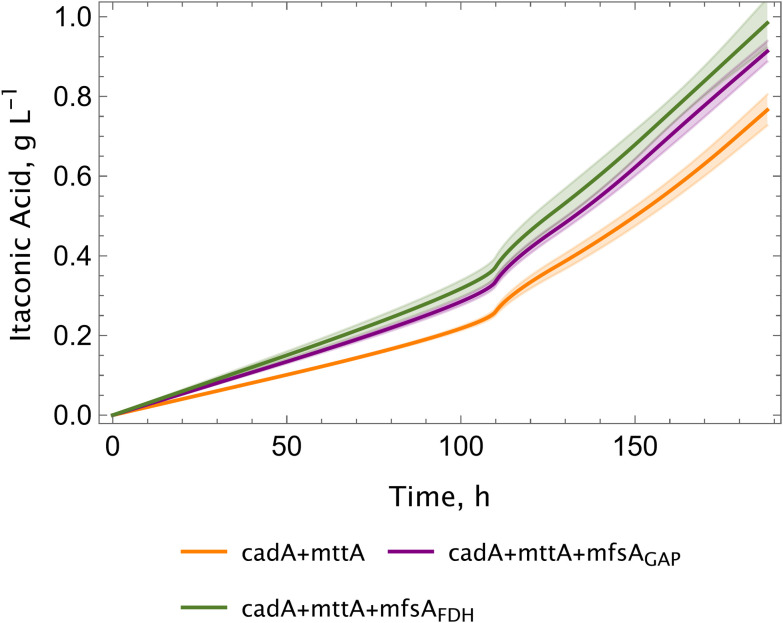
Co-expression of *mfsA* improves the itaconic acid production. Screening was performed in shake flasks at 25–28 °C, 5% CO_2_. Seven biological replicates for each construct were screened and standard deviations (±) were calculated and shown in shades.

The co-expression of *mfsA* resulted in a 20% increase in the final itaconic acid titers, yielding approximately 1 g L^−1^ in both the cadA + mttA + mfsA_FDH1_ and cadA + mttA + mfsA_GAP_ strains (Fig. 5). Growth was not affected in any of the strains (0.005 h^−1^), specific productivity was similar and varied between 3.1–3.4 mg g^−1^ h^−1^ (Table 1). For further experiments, a representative clone for cadA + mttA + mfsA_GAP_ was selected based on the advantages offered by a constitutive promoter for an exporter.

**Fig. 6:**
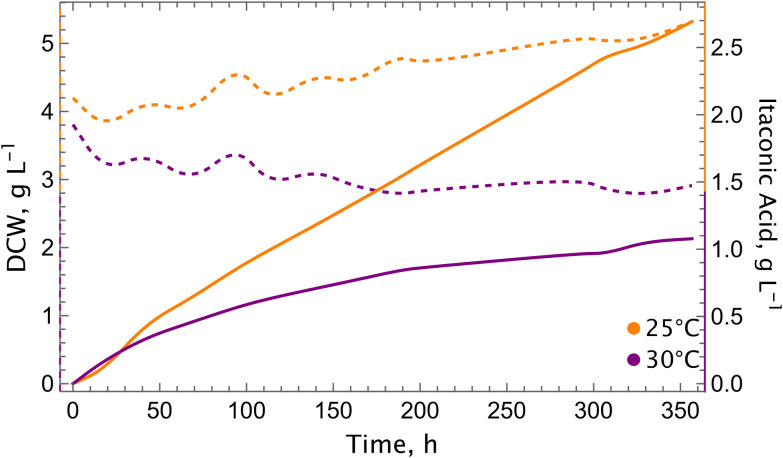
Elevated CO_2_ concentration and decreased temperature improves the titers of itaconic acid production with the cadA + mttA + mfsA_GAP_ strain in bioreactor cultivation. Growth (dashed lines) and itaconic acid production (solid lines) profiles at 25 °C and 30 °C are shown. Experiments were conducted in single runs in lab-scale bioreactors, using 10% CO_2_ in the inlet gas.

After verifying the superior performance of the strain with co-expression of *mfsA*, we tested its performance in bioreactor cultivations. We included a bioreactor run at 30 °C to compare with 25 °C, thereby investigating the potential benefits of elevated temperatures on itaconic acid production and export (Fig. 6). This has been reported recently by Severinsen *et al.*^24^ where 30–32 °C were reported as optimum temperatures for itaconic acid production by *K. phaffii* on methanol. However, in our experimental setting, 25 °C was found to be more conducive than 30 °C, with the itaconic acid titer reaching 2.70 g L^−1^ after a 360-hour cultivation period (Fig. 6). In alignment with our previous bioreactor cultivations conducted at 30 °C, the cadA + mttA + mfsA_GAP_ strain did not demonstrate any signs of growth. However, the specific growth rate of the same strain was 0.0007 h^−1^ at 25 °C, which is 1.75-fold higher than the strain cadA + mttA (Fig. 4), while the specific productivity was 1.2-fold higher (1.59 *vs.* 1.32 mg g^−1^ h^−1^).

### Improving efficiency of the CBB cycle increases itaconic acid production

Itaconic acid is a by-product of the TCA cycle, and its production is positively correlated with growth. As was demonstrated above, lowering the temperature can enhance growth. Therefore, we hypothesized that a strain capable of enhanced growth might increase productivity. To test the hypothesis, we constructed a production strain, MC-CBB_cadA + mttA + mfsA_GAP_, using a parental strain that carries multiple copies of RuBisCO and chaperones.^26^ This strain showed superior growth compared to the single-copy strain, that we have employed to construct production strains until this point. The performance of MC-CBB_cadA + mttA + mfsA_GAP_, in addition to the multicopy control strain, was evaluated in shake flask experiments ( Fig. S3).

As anticipated, the MC-CBB_cadA + mttA + mfsA_GAP_ strain exhibited a titer of 1.37 g L^−1^ itaconic acid at 195 hours (equivalent to 2.02 g L^−1^ at 262 hours) during the cultivation period, while the cadA + mttA + mfsA_GAP_ strain reached to a titer of 1.11 g L^−1^ (1.57 g L^−1^ at 262 hours). The productivity of the MC-CBB_cadA + mttA + mfsA_GAP_ strain was found to be 4.15 mg g^−1^ h^−1^, which is a 1.3-fold increase in comparison with the cadA + mttA + mfsA_GAP_ strain (Table 1).

### Increasing the gene copy number of *mfsA* improves the titers

**Fig. 7:**
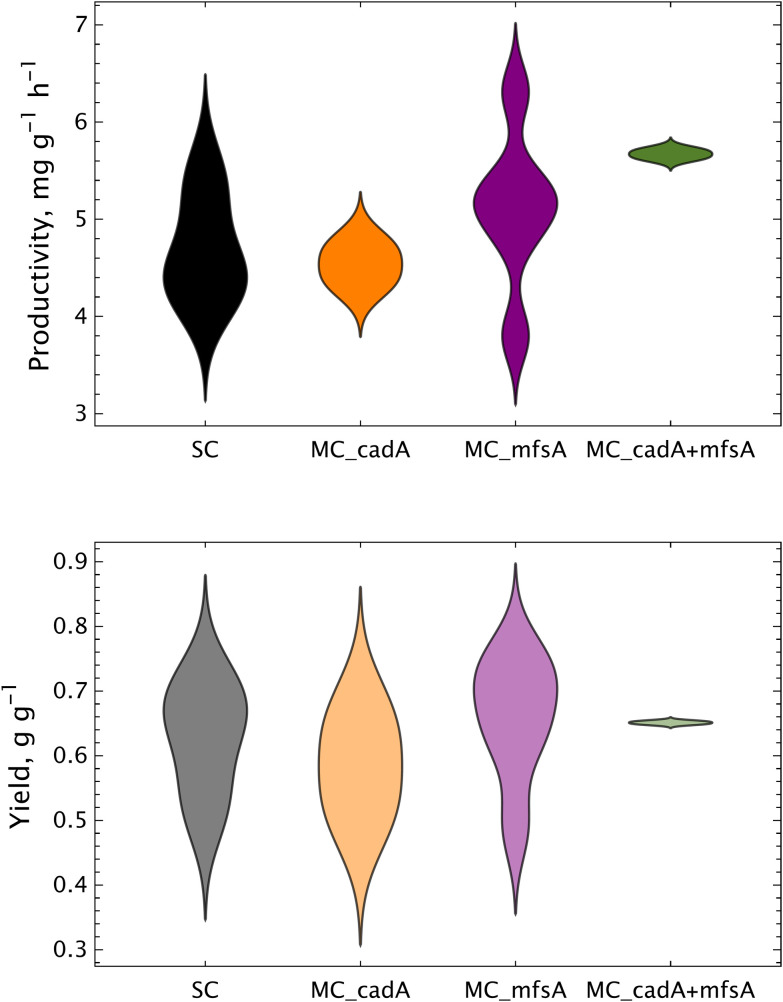
Increasing the copy number of *mfsA* enhances the itaconic acid productivity (mg IA g DCW^−1^ h^−1^) and yield (g IA g DCW^−1^). At least 2 clones from each construct were screened. Screening was performed in shake flasks at 25 °C, 5% CO_2_. SC: single copy, MC: multi copy.

After demonstrating that the MC-CBB_cadA + mttA + mfsA_GAP_ strain has a superior performance in the production of itaconic acid, we decided to increase the gene copy number of the itaconic acid metabolism to test whether multiple copies could improve the titers further. Here, either we integrated multiple copies of each gene individually (*cadA*, *mttA* or *mfsA*) or combinations of these genes to construct MC-CBB-IA_cadA + mttA + mfsA_GAP_ strains. We selected random colonies from each transformation and screened them for the final itaconic titers and respective product yields and productivities during the autotrophic growth (Fig. 7 and Table S1).

The integration of *mttA* alone resulted in a substantial growth impairment. This finding is consistent with our previous results, where *mttA* was co-expressed with a strong promoter, resulting in impaired growth.^16^

The co-expression of *cadA* alone did not result in a significant enhancement of production and the best results were achieved when multiple copies of *mfsA* were present. This is corroborated by gene copy number (GCN) analysis (Table S1). The enhanced levels of itaconic acid titers, product yields and productivities (g g^−1^ DCW) were consistent with the elevated copies of *mfsA*, exhibiting a specific productivity of 5.11 mg g^−1^ h^−1^ (Table 1). Combination of *mfsA* with *cadA* led to a slight improvement in the productivity, while the yields were similar. Consequently, the clone from the MC-CBB-IA_cadA + mttA + mfsA_GAP_ strain with the highest productivity, which contains around six copies of *mfsA*, was selected for further bioreactor experiments (Table S1).

### Bioreactor performance improves with shake flask-mimicking complex medium batch phase

Following the construction of the MC-CBB-IA_cadA + mttA + mfsA_GAP_ strain, bioreactor cultivations were performed utilising the parameters that had been determined in the preceding experiments: 25 °C, 10% CO_2_ and 16% dissolved oxygen concentration. However, during bioreactor cultivations, it was still not possible to achieve or exceed the titers and productivity levels obtained in shake flasks. Despite the fact that temperature and dissolved oxygen levels were identified as two pivotal parameters, these efforts were unsuccessful, thus pointing out a limitation.

Our standard bioreactor cultivations included a glycerol batch phase with yeast nitrogen base (YNB) containing 8 g L^−1^ glycerol. In this setup, cells were grown to a DCW of approximately 4 g L^−1^ (OD 20) and the autotrophic growth conditions for the itaconic acid production were initiated with 10% CO_2_. This constitutes a fundamental difference between shake flask and bioreactor cultivations, wherein a preculture in YPG is employed in the shake flasks, as opposed to the YNB + Glycerol batch phase in the bioreactor. In order to address the aforementioned problem, we sought to mimic the conditions of the shake flask screenings in a lab-scale bioreactor cultivation as closely as possible: we employed another approach, where a “batch phase” was performed in shake flasks in YPG instead of YNB + Glycerol, similar to shake flask cultivations. After growing the cells in YPG, we washed them twice to remove the residuals and inoculated the bioreactor with an OD of approximately 20, and the autotrophic production phase with 10% CO_2_ was initiated.

**Fig. 8:**
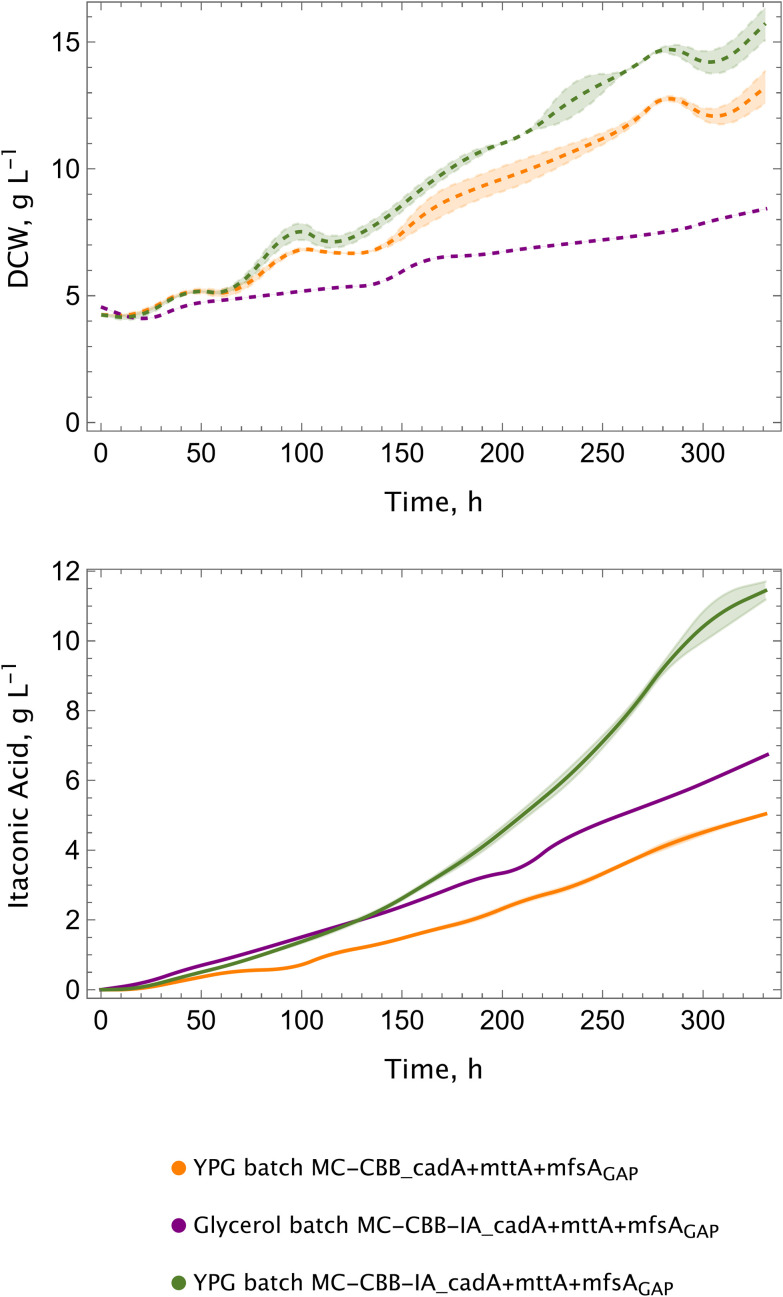
Preculture medium affects the performance of the itaconic acid production strains. Growth (dashed lines) and itaconic acid production (solid lines) profiles. Experiments were conducted at 25 °C in duplicates in lab-scale bioreactors, using 10% CO_2_ in the inlet gas.

The analysis of the growth profile revealed the beneficial effect of incorporating a “batch phase” in a complex medium, as it enabled the cells to reach higher biomass titers with a growth rate of 0.004 h^−1^ compared to 0.002 h^−1^ (Fig. 8 and Table 1). The final titers achieved under these conditions were the highest among the tested conditions and measured as 11.84 g L^−1^. This is consistent with the observed continuous growth, as itaconic acid is a product of the primary metabolism. The results demonstrate a clear enhancement in itaconic acid production for the MC-CBB-IA_cadA + mttA + mfsA_GAP_ strain, with a productivity of 3.94 mg g^−1^ h^−1^. This is a 2-fold increase compared to the control strain (MC-CBB_cadA + mttA + mfsA_GAP_) in YPG batch culture and 1.2-fold higher than the same strain grown in a YNB + Glycerol batch (Table 1).

## Discussion

In this study, we integrate metabolic engineering strategies to optimize flux distribution in heterologous pathways with further bioprocess design focusing on the key process parameters, *i.e.* DO and temperature, to enhance itaconic acid production *via* CO_2_ conversion in bioreactor cultivation.

Previously, we demonstrated that a DO concentration of 8% enhanced itaconic acid production when compared to levels of 20%.^16^ Therefore, here, the investigation was expanded to encompass DO values below 20%, and we included the DO concentrations of 4%, 8%, and 16%, to examine the relationship between oxygen levels and itaconic acid production. The impact of oxygen should be considered at two levels, namely the oxygenation side reaction of RuBisCO and the methanol oxidation involved in the dissimilation. Increasing oxygen availability may potentially decrease the reaction rates of RubisCO on CO_2_ as oxygen serves as an additional substrate, therefore lower oxygen concentrations could be advantageous. However, the cells require oxygen for the methanol oxidation and *K. phaffii* is unable to grow anaerobically. Consequently, a reduction in oxygen availability may lead to a decrease in reducing equivalents, which are produced during the methanol dissimilation process. The lack of significant changes in the transcriptional levels of key genes associated with the CBB cycle and itaconic acid metabolism suggests that regulation might occur at the post-transcriptional or metabolic level. Despite unchanged transcript levels, a decrease in RuBisCO's CO_2_/O_2_ specificity and reduced carboxylation rates at elevated oxygen concentrations could lead to lower flux through the CBB cycle.^27^ In our previous work, we demonstrated that higher oxygen levels shift the balance of the RuBisCO reaction toward oxygenation, resulting in increased phosphoglycolate formation. This shift reduces the overall carboxylation activity of the CBB cycle in synthetic autotrophic *K. phaffii.*^28^ In this context, a DO level of 16% was found to be the optimum oxygen level with a 1.4-fold increase in itaconic acid production titer.

The second key parameter is the temperature, which had a substantial impact both on production and growth, when it was switched from 30 °C to 25 °C. Previously, we evaluated different initial biomass values to see their effect on itaconic production at 30 °C.^16^ In this setup, even though the production was improved, there was almost no growth; especially when the initial biomass was higher. Conversely, at 25 °C, cells exhibited growth, suggesting a more efficient CBB cycle compared to 30 °C. Furthermore, we examined the impact of temperature on bioreactor cultivations, where the itaconic acid titer reached 1.39 g L^−1^ at 25 °C, representing a 2.1-fold increase compared to 30 °C. The benefit of lower temperature was also reflected in the gene expression levels of the CBB cycle, as at lower temperatures, RuBisCO and PRK were more highly expressed compared to 30 °C. One evident reason for obtaining higher titers and better growth at lower temperature could be the higher gas solubility in the aqueous media. The availability of CO_2_ might have been increased due to the lower temperature, which then leads to an increased carbon flux in the CBB cycle. A secondary rationale pertains to the catalytic properties of RuBisCO, as shown by Yamori *et al.* (2006).^29^ They demonstrated that as temperature rises, the carboxylation rate and specificity factor (*S*_c/o_) decrease. This phenomenon further elucidates why lower temperatures yield superior titers and promote growth.

In our previous study, we demonstrated that a balanced co-expression of *mttA*, a mitochondrial membrane transporter, is essential for enhanced production and growth. Here, we further demonstrate the role of another transporter, *mfsA*, leading to higher productivity, confirming previous findings.^24,30^ The enhanced titers observed in the *mfsA*-expressing strain are probably indicative of a bottleneck in the secretion of itaconic acid, rather than a deficiency in its production. In fact, increasing the copy number of *mfsA* led to a substantial enhancement in itaconic acid production, thereby enhancing the pull effect. Conversely, the introduction of additional copies of cadA or mttA did not result in a significant change in itaconic acid levels, whereas higher copy numbers of mttA even led to a substantial growth impairment. This outcome is consistent with prior findings.^16,24^ The enhanced expression of mttA is likely to cause a depletion of *cis*-aconitate, an intermediate in the TCA cycle, leading to diminished TCA cycle activity. Consequently, it is essential to balance the expression of heterologous genes, particularly when they interfere with native essential metabolic pathways, to ensure efficient product synthesis.

The results of integrating multiple copies of the CBB cycle and itaconic acid metabolism genes, showed that it is crucial to optimize the balance of carbon flux from the CBB cycle to biomass and itaconic acid production precursors. Increasing the copy numbers of the pivotal gene, RuBisCO, and its chaperones, GroEL and GroES, not only improved the growth but also led to a substantial increase in the production performance of the synthetic autotrophic strain. In a previous study, adaptive evolution led to mutations in PRK with reduced expression levels and enzyme activity, and reverse engineering that in the initial CBB cycle strain confirmed increased growth relative to the parent strain.^31^ However, it was observed that this strain did not exhibit superiority in terms of itaconic acid production, despite its enhanced growth capacity.^16^ To investigate whether increasing the copies of *mfsA* would lead to a further improvement with a pull effect, the previously mentioned reverse engineered strain was used to integrate itaconic acid metabolism with additional copies of *mfsA*. While higher titers were achieved due to faster growth, the specific productivity of the reverse engineered strain was lower than that of the parent strain, (MC_IA + cadA_mttA_mfsA_GAP_) ( Table S2). These findings indicate that increasing the growth rate does not necessarily increase the productivity and that a balanced carbon flux between growth and production is essential. The significance of a balanced metabolism is further underscored by the integration of multiple copies of *cadA*, *mttA*, or *mfsA* from the itaconic acid metabolism. The integration of *mfsA*, accompanied by *cadA*, exhibited the highest productivity and titer, while the introduction of additional copies of *cadA* alone did not enhance the strain's performance.

Although optimization of the temperature and dissolved oxygen concentration enhanced itaconic acid production, both the yield and productivity were initially lower in bioreactor cultivations compared to shake flask experiments. Key differences between the two cultivation systems include pH control in bioreactors—where the pH was maintained at 6.0, whereas in shake flasks it dropped to approximately 4.0–4.5 by the end of cultivation—as well as differences in dissolved gas concentrations. In bioreactors, dissolved CO_2_ and O_2_ levels were monitored and controlled, with the dissolved CO_2_ concentration measured at 8.9 ± 0.3%, whereas no such control was present in shake flask cultures. Notably, ethanol production by *K. phaffii* has also been reported in glucose-based shake flask cultivations, which suggests oxygen-limited conditions, as *K. phaffii* is known to produce ethanol under reduced O_2_ availability.^32,33^ This indicates that mass transfer limitation likely occurred more in shake flasks rather than in bioreactors, making mass transfer limitation in the latter an unlikely explanation for the lower performance. A major distinction between the two systems was the glycerol batch phase in synthetic medium for bioreactors *versus* in complex medium containing yeast extract and peptone in shake flasks. Initiating the production phase using the cells from a complex medium may have replenished intracellular amino acid pools essential for growth and protein synthesis, potentially enhancing cellular performance.^34^ Additionally, since cyclic metabolic pathways often require extended lag phases for full activation, the nutrient-rich conditions in complex medium may have supported the production of key intermediates during the batch, thereby improving the efficiency of the subsequent production phase.^33^ Finally, we calculated the key process parameters of the bioreactor cultivation, where an itaconic acid titer of approximately 12 g L^−1^ was reached with the highest specific productivity among the tested conditions in bioreactors (3.94 mg g^−1^ h^−1^) with a space time yield of 0.04 g L^−1^ h^−1^ (Table 1). Itaconic acid production from C1 carbon sources has so far only been demonstrated by Severinsen *et al.*^24^ In fed-batch cultivations with methanol as the carbon source, they reported a specific productivity of 10 mg g DCW^−1^ h^−1^, a product yield of 0.22 g g DCW^−1^, and a space time yield of 0.49 g L^−1^ h^−1^ resulting in a final itaconic acid titer of 55.4 g L^−1^.

Total product yield on methanol, that was used for NADH production *via* the dissimilation, was 0.09 g g^−1^ while the net CO_2_ production was 0.25 mmol CO_2_ g^−1^ h^−1^. Overall, 52 g MeOH was utilized during the cultivation, while 37 g CO_2_, 5 g biomass and 5 g itaconic acid were produced. A total of 10.6 C-mol (1.6 C-mol from methanol and 8.9 C-mol from CO_2_) was supplied to the system throughout the cultivation, while 10.2 C-mol (0.2 C-mol as biomass, 0.2 C-mol as itaconic acid, and 9.8 C-mol as CO_2_) was accounted for in the output, corresponding to a carbon recovery of 96%. The unaccounted carbon may result from measurement inaccuracies, methanol evaporation, or the formation of by-products not detected by HPLC. Considering that 1.6 C-mol of methanol are oxidized to 1.6 C-mol of CO_2_*via* the methanol dissimilation pathway for energy production, the remaining 8.1 C-mol of carbon leaving the system must have originated from the fed CO_2_. This indicates that, out of the 8.9 C-mol of CO_2_ supplied, 0.8 C-mol were assimilated into biomass and itaconic acid, resulting in a carbon conversion efficiency of 0.5 C-mol C-mol^−1^ (0.24 C-mol C-mol^−1^ for itaconic acid and 0.26 C-mol C-mol^−1^ for biomass, respectively).

These calculations show that the bioprocess is still CO_2_-positive and several scale-up challenges remain. Key limitations including slow cellular growth, low space–time yields and productivities must be addressed to enhance process efficiency, *via* rational engineering or adaptive laboratory evolution. Due to the inherently low solubility of CO_2_ in aqueous systems, pressurized bioreactor operation may enhance CO_2_ availability, thereby improving both growth and product formation. Additionally, CO_2_-based bioprocesses require a sustainable energy source; thus, the use of renewable electricity or the electrochemical conversion of CO_2_ into energy-rich intermediates such as methanol or formate must be both efficient and economically viable. Considering a process in which methanol is produced *via* the electrochemical reduction of CO_2_, there is significant potential for itaconic acid production with a synthetic autotrophic yeast strain to become a CO_2_-negative process. A key step there would be to engineer the methanol dissimilation pathway by replacing alcohol oxidase (AOX) with an alcohol dehydrogenase (ADH). This modification would lead to the production of an extra mole of NADH, thereby reducing the methanol requirement by 33% and contributing to the achievement of CO_2_-neutral or negative bioprocesses.^35^ The theoretical yield per MeOH is 0.77 mol fixed C-mol mol^−1^ MeOH with AOX, while it is 1.15 mol fixed C-mol mol^−1^ MeOH with ADH^36^ which means that in the latter case with ADH the use of our strains can become carbon negative. As noted above, in the present case with AOX we reach 33% of the theoretical yield (0.25 mol fixed C-mol^−1^ MeOH), a value that provides a perspective for further strain and process development.

## Conclusion

In this study, we demonstrated that a synthetic autotrophic *K. phaffii* is able to produce itaconic acid by the direct conversion of CO_2_, achieving final titers of approximately 12 g L^−1^ in the bioreactor cultivations. We showed that balancing the host metabolism considering the interplay of the heterologous metabolism with the native metabolic pathways is essential to reach higher production performance. Additionally, beyond metabolic engineering, precise bioprocess design was one of the key steps in enhancing productivity.

Conventional itaconic acid production by filamentous fungi such as *A. terreus* or *U. maydis* relies on glucose-based processes. Thus, the utilization of C1 carbon sources as a feedstock holds significant potential for enabling more sustainable itaconic acid production, despite current titers remaining lower than those achieved with native glucose-based processes.^37–39^ Considering the key process parameters, the current status of our CO_2_-based process is not yet able to compete with the traditional fermentation processes, where first- or second-generation feedstocks are used. However, we believe our results highlight the potential of single-carbon substrate-based processes, utilizing CO_2_ directly or in combination with other CO_2_-derived substrates like methanol or formate, to develop CO_2_-neutral or negative bioprocesses in line with future sustainability goals.

## Methods

### Construction of the strains

The synthetic autotrophic *K. phaffii* strain described by Gassler *et al.* (2020)^22^ was used as the host to construct the organic acid producing strains. The construction of the strain carrying *cadA* and *mttA* was described by Baumschabl *et al.* (2022).^16^ The *mfsA* gene encoding a cytoplasmic itaconic acid exporter was inserted into the *GUT1* locus by CRISPR-Cas9^40^ in the cadA + mttA strain. Two different promoters (pFDH1 and pGAP) were tested to find the optimal expression of *mfsA*. Plasmid constructions and transformations were performed by Golden Gate Assembly (GGA) and the CRISPR-Cas9 system described in Gassler *et al.* (2019).^31^ Integration to the correct loci was verified by PCR.

Multiple copies of *cadA, mttA, mfsA*, along with combinations of these genes and RuBisCO and its chaperones were performed as described by Severinsen *et al.* (2024).^24^ A list of the strains used in this study is given in Table 2.

**Table 2:** *K. phaffii* strains used in this study

Strain	Genotype	References
Control	CBS7435 ΔAOX1::pAOX1_TDH3 + pFDH1_PRK + pALD4_PGK1 ΔDAS1::pDAS1_RuBisCO + pPDC1_groEL + pPP1B_groES ΔDAS2::pDAS2_TLK1 + pRPS2_TPI1	Gassler *et al.* (2020)^22^
cadA	Control + pAOX1_cadA	Baumschabl *et al.* (2022)^16^
cadA + mttA	Control + pAOX1_cadA + pPOR1_mttA	Baumschabl *et al.* (2022)^16^
cadA + mttA + mfsA_FDH1_	Control + pAOX1_cadA + pPOR1_mttA + pFDH1_mfsA	This study
cadA + mttA + mfsA_GAP_	Control + pAOX1_cadA + pPOR1_mttA + pGAP_mfsA	This study
MC-CBB_cadA + mttA + mfsA_GAP_	Control + Multicopy CBB^26^ + pAOX1_cadA + pPOR1_mttA + pGAP_mfsA	This study
MC-IA_cadA + mttA + mfsA_GAP_	Control + Multicopy itaconic acid + pAOX1_cadA + pPOR1_mttA + pGAP_mfsA	This study
MC-CBB-IA_cadA + mttA + mfsA_GAP_	Control + Multicopy CBB^26^ – itaconic acid + pAOX1_cadA + pPOR1_mttA + pGAP_mfsA	This study
RE_cadA + mttA	Reverse engineered control^31^ + pAOX1_cadA + pPOR1_mttA +	This study
RE_MC_IA_cadA + mttA + mfsA	Reverse engineered control^31^ + Multicopy itaconic acid + pAOX1_cadA + pPOR1_mttA + pGAP_mfsA	This study

### Shake flask cultivations

Shake flask cultivations were carried out in 100 mL narrow-neck flasks with cotton caps at 25 or 30 °C with 5 or 10% constant CO_2_ supply, shaken at 180 rpm in 22 mL buffered YNB (3.4 g L^−1^, pH 6) supplemented with 10 g L^−1^ (NH_4_)_2_SO_4_ as the nitrogen source. Cells were inoculated with the target starting OD_600_ (4 or 20) and induced with 0.5% (v/v) methanol at the start of the main culture, and adjusted to 1% (v/v) methanol after the first sampling until the end of the cultivations. Screenings were performed for 192 h (8 days). Cell growth (OD_600_) was monitored during the cultivation and extracellular metabolite concentrations (methanol and itaconic acid) were measured by high-performance liquid chromatography (HPLC) and culture volume was corrected for evaporation by the addition of water.

### Bioreactor cultivations

Bioreactor cultivations were conducted in 1.4 L DASGIP reactors (Eppendorf). pH was kept at 6.0 by using 2 mol L^−1^ NaOH or 5 mol L^−1^ KOH. Dissolved oxygen (DO) concentration was controlled by adjusting the inlet oxygen concentration, stirrer speed and inlet gas flow whereby 5%, 200 rpm and 6 sL h^−1^ were the minimal setpoints, respectively. DO was set to 4, 8, or 16% to explore the effect of oxygen concentration on growth and itaconic acid production. The autotrophic cultivation was performed from beginning on using 10% CO_2_ in the inlet gas and 0.5% methanol. After the first sample (appr. 16 h) methanol concentration was adjusted to 1%. Cultivations were conducted using YNB media supplemented with 10 g L^−1^ (NH_4_)_2_SO_4_ as the nitrogen source and buffered using 100 mmol L^−1^ phosphate buffer at pH 6. Temperature was set either to 25 or 30 °C. Bioreactor cultivations were performed for 250 h+ to assess the longer term production performance of the strains in larger scale.

The bioreactor cultivations were performed in two different experimental setups: (1) A YPG preculture was inoculated to an OD of 1 in YNB media including 8 g L^−1^ glycerol. The batch phase was performed to reach an end biomass of approximately OD_600_ of 20 (*ca.* 4 g L^−1^), based on a yield on glycerol of approximately 0.5. Following the batch end, cultures were induced with 0.5% (v/v) methanol and provided with constant supply of 10% CO_2_ in the inlet gas starting the autotrophic cultivation. (2) A YPG preculture was inoculated to an OD_600_ of 20 and the autotrophic process conditions were directly initiated, skipping the batch phase on YNB with glycerol.

In both scenarios, methanol concentration was adjusted to 1% at the first sample points (after appr. 16 h). From this time on samples were taken daily including OD, dry cell weight (DCW), and HPLC samples. After each sampling the methanol concentration was adjusted to 1% (v/v). Overall specific growth rate, production rates and product yields of the growing strains in the shake flasks and bioreactor cultivations during the autotrophic production phase were calculated according to eqn (1)–(3):1
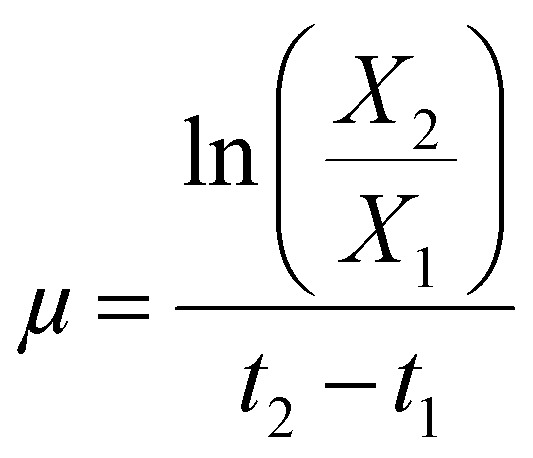
2
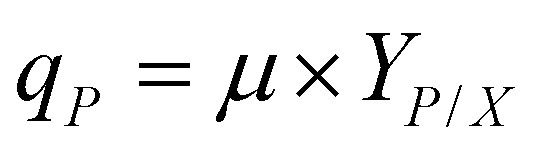
3
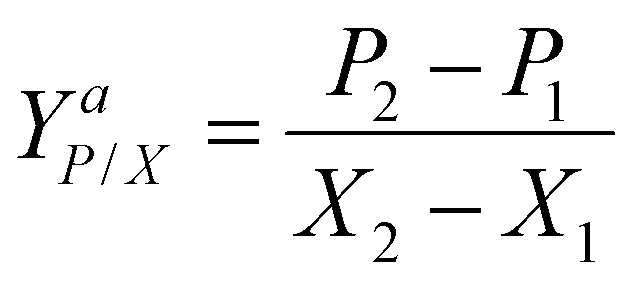
*μ* is the specific growth rate (h^−1^), *X* is the cell concentration (g L^−1^) at *t* (h) of the cultivation, *P* is the product concentration (mg L^−1^) at *t* (h) of the cultivation, *q*_*P*_ is the specific production rate (mg g^−1^ h^−1^), *Y*^*a*^_*P*/*X*_ is the product yield per biomass (mg g^−1^) of growing strains.

For the nongrowing strains, production rates and yields during the autotrophic production phase were calculated according to eqn (4) and (5):4
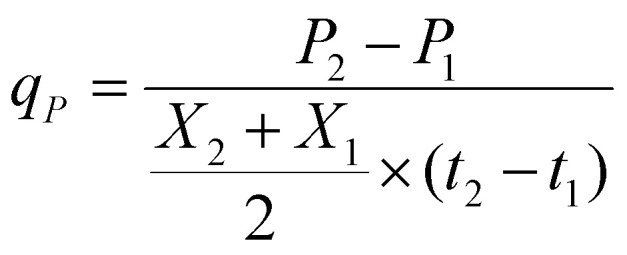
5
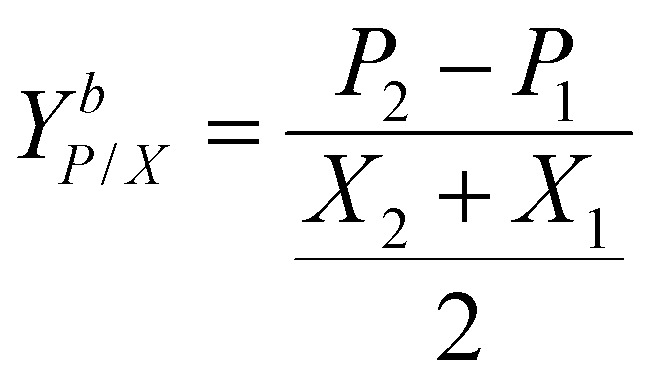
*μ* is the specific growth rate (h^−1^), *X* is the cell concentration (g L^−1^) at *t* (h) of the cultivation, *P* is the product concentration (mg L^−1^) at *t* (h) of the cultivation, *q*_*P*_ is the specific production rate (mg g^−1^ h^−1^), *Y*^*b*^_*P*/*X*_ is the product yield per biomass (mg g^−1^) of non-growing strains.

### Dry cell weight (DCW) measurements

For dry cell weight measurement, a known volume of bioreactor cultivation broth was centrifuged and pellets were washed twice with distilled water. Washed samples were dried in preweighed beakers at 100 °C for 24 h.

### HPLC measurements

HPLC measurements were performed by using a Biorad Aminex HPX-87H HPLC column (300 × 7.8 mm).^16^ Samples were centrifuged at 4000*g* for 10 min, and the supernatant of each sample was mixed with 40 mM H_2_SO_4_ resulting in a final concentration of 4 mM. Samples were vortexed and centrifuged at full speed (16 100*g*) for 5 min at room temperature. Following the centrifugation, they were filtered using a 0.22 μm filter into the vials for the HPLC analysis.

### Genomic DNA extraction and gene copy number analysis

Genomic DNA (gDNA) of the *K. phaffii* strains was extracted from overnight cultures using the Wizard Genomic DNA purification kit (Promega Corp., USA) according to the manufacturer's instructions. The quality, purity and concentration of the isolated gDNA was verified with Nanodrop.

Following the genomic DNA extraction, an RT-PCR was carried out using 2× qPCR S'Green BlueMix (Biozym Blue S'Green qPCR Kit). gDNA was mixed (2.7 ng μL^−1^ in 3 μL) with respective primers (*cadA, mttA, mfsA*; 0.4 μL from 10 mM stocks), 2× qPCR S'Green BlueMix (5 μL) and water (up to 10 μL). All samples were analyzed in triplicates with non-template controls. The copy numbers of the respective genes were estimated in comparison to the copy number of the parent strain using the ΔΔCT method.^41^

## Author contributions

ÖA designed and performed the construction of strains and their evaluation. MB performed the construction of strains. LL supported cloning, cultivation and analysis of strains. DM conceived of the study and supervised the project with ÖA. ÖA and DM wrote the manuscript with support by all authors. All authors read and approved the final manuscript.

## Conflicts of interest

There are no conflicts of interest to declare.

## Supplementary Material

GC-027-D5GC03149D-s001

## Data Availability

Data for this article, including the figures in the manuscript are available at Figshare at https://doi.org/10.6084/m9.figshare.29370929. The data supporting this article have been included as part of the SI. See DOI: https://doi.org/10.1039/d5gc03149d.
