# Supplementary material for: Synthetic autotrophic yeast enables high itaconic acid production from CO2via integrated pathway and process design
Source: Green Chem. 2025 Sep 24;27(40):12715–26. doi: 10.1039/d5gc03149d (PMC12459085; doi:10.1039/d5gc03149d)
Supplement: GC-027-D5GC03149D-s001 [file GC-027-D5GC03149D-s001.pdf]

Supplementary Information (SI) for Green Chemistry.
This journal is © The Royal Society of Chemistry 2025

Supplementary File

Synthetic autotrophic yeast enables high itaconic acid production from CO_2 via integrated pathway and process design

Özge Ata[1,2,*], Lisa Lutz[1,2], Michael Baumschabl [1,2], Diethard Mattanovich[1,2]

[1] BOKU University, Vienna, Institute of Microbiology and Microbial Biotechnology, Department of Biotechnology and Food Science, 1190 Vienna, Austria

[2] Austrian Centre of Industrial Biotechnology, Vienna, 1190, Austria

* Corresponding author: oezge.ata@boku.ac.at

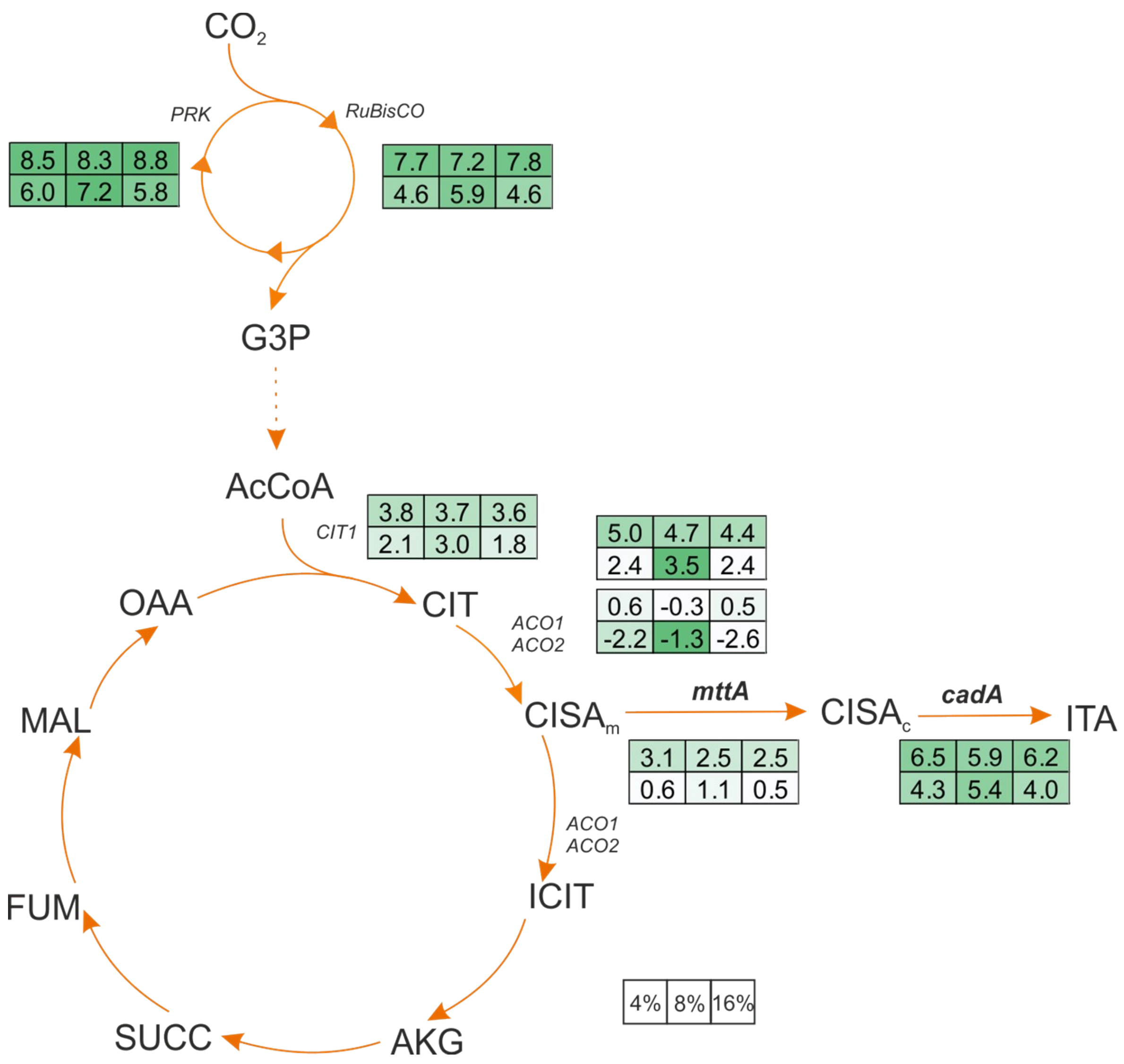

Supplementary Figure 1. Gene expression levels of the cadA+mttA strain at different oxygenation conditions at 30°C in the bioreactor cultivation using 5% CO_2 in the inlet gas stream. log2-fold change values in the gene expression levels are given. All expression levels are normalized against the strain's own actin (*ACT1*) expression. First and second row values belong to 103 h and 198 h samples, respectively. First, second, and third column values belong to the cultivations performed at 4%, 8% and 16% dissolved oxygen concentration, respectively. AcCoA: acetyl-coenzyme A, AKG: alpha-ketoglutarate, *cadA*: *cis*-aconitate decarboxylase, $CISA_c$: cytosolic *cis*-aconitate, $CISA_m$: mitochondrial *cis*-aconitate, CIT: citrate, ICIT: isocitrate, FUM: fumarate, ITA: itaconic acid, MAL: malate, *mttA*: mitochondrial tricarboxylic acid transporter, OAA: oxaloacetate, *PRK*: phosphoribulokinase, *RuBisCO*: ribulose 1,5-bisphosphate carboxylase/oxygenase, SUCC: succinate, peroxisome. c: cytosol, m: mitochondria.

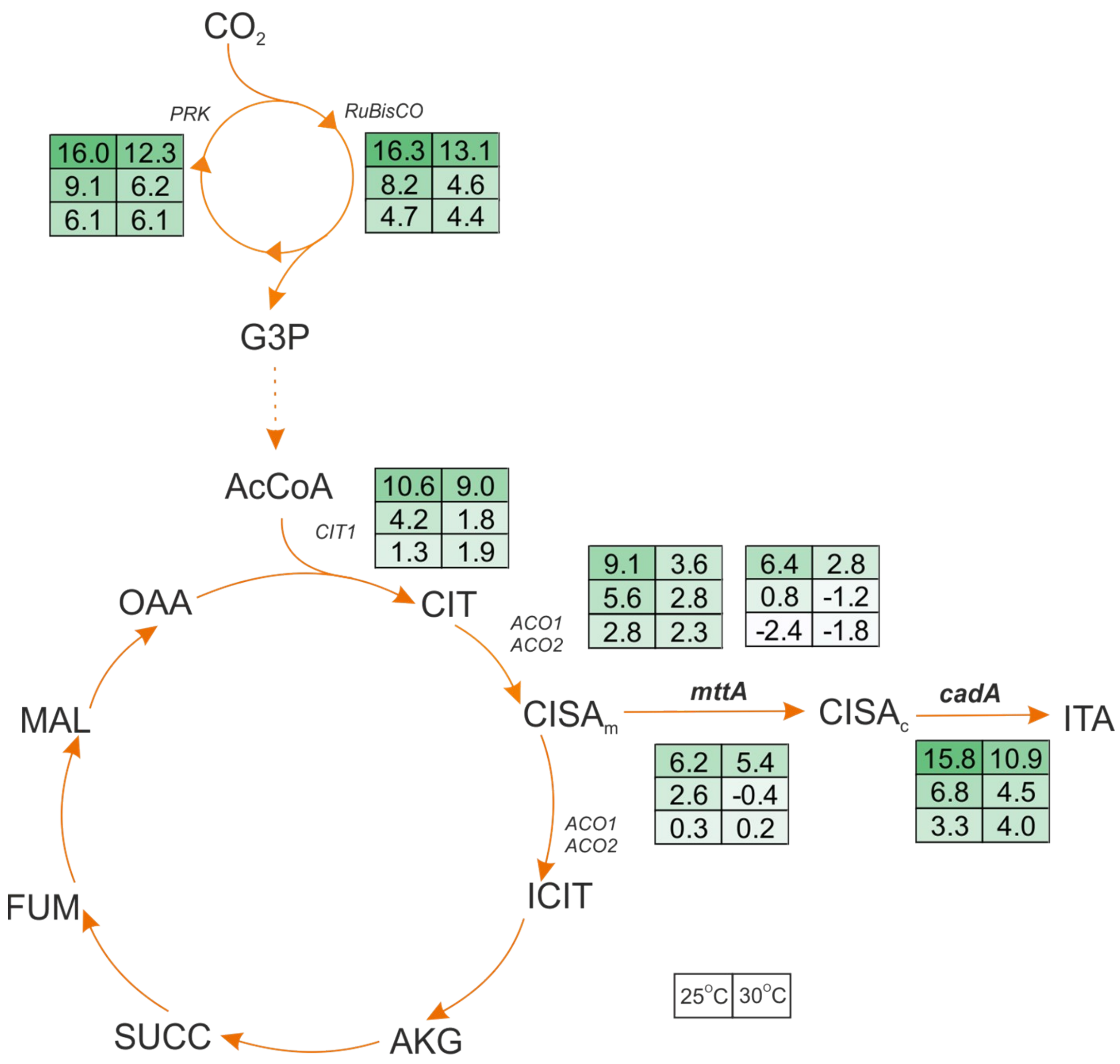

Supplementary Figure 2. Gene expression levels at different temperatures of the cadA+mttA strain at 25°C and 30°C in the bioreactor cultivation using 10% CO_2 in the inlet gas stream. log2-fold change values in the gene expression levels are given. All expression levels are normalized against the strain's own actin (*ACT1*) expression. First, second and third row values belong to 27 h, 148 h, and 194 h samples, respectively. Left and right column values belong to the cultivations at 25 and 30°C, respectively. AcCoA: acetyl-coenzyme A, AKG: alpha-ketoglutarate, *cadA*: *cis*-aconitate decarboxylase, $CISA_c$: cytosolic *cis*-aconitate, $CISA_m$: mitochondrial *cis*-aconitate, CIT: citrate, ICIT: isocitrate, FUM: fumarate, ITA: itaconic acid, MAL: malate, *mttA*: mitochondrial tricarboxylic acid transporter, OAA: oxaloacetate, *PRK*: phosphoribulokinase, *RuBisCO*: ribulose 1,5-bisphosphate carboxylase/oxygenase, SUCC: succinate, peroxisome. c: cytosol, m: mitochondria.

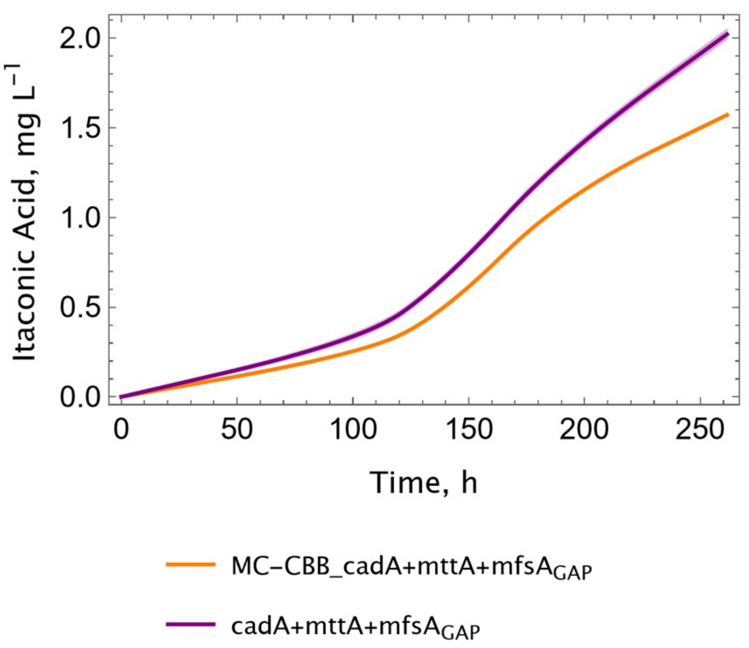

Supplementary Figure 3. Higher copy number of the CBB cycle improves itaconic acid production. Itaconic acid production profile of the cadA+mttA+mfsA$_{GAP}$ and MC-CBB_cadA+mttA+mfsA$_{GAP}$ strains. Screening was performed at shake flask at 25°C, with 5% CO_2. Four biological replicates for each construct were screened and standard deviations (±) were given in shades.

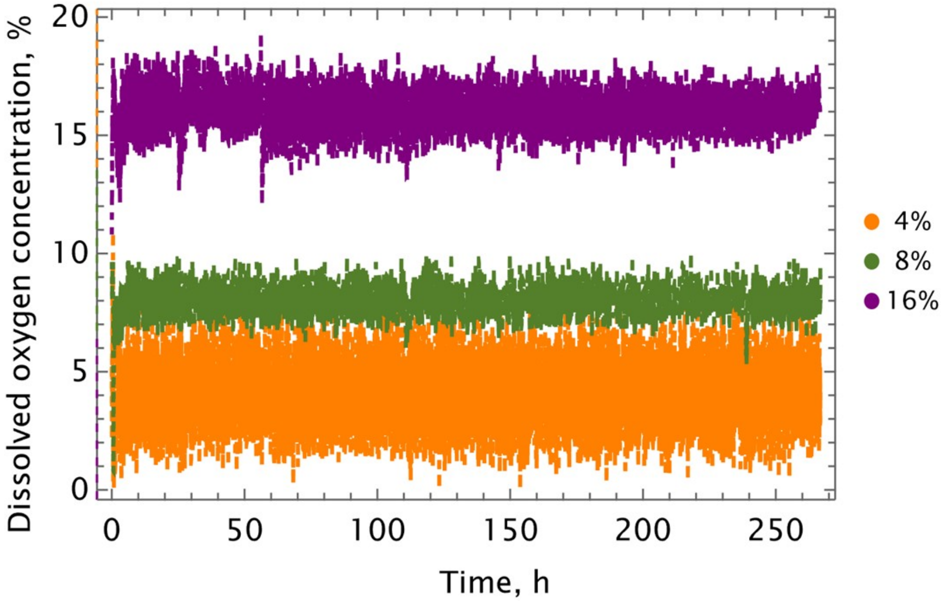

Supplementary Figure 4. Dissolved oxygen profiles throughout the cultivation. Cultivation was performed in bioreactors at 30°C, with 5% CO_2.

Supplementary Table 1. Growth, itaconic acid production characteristics and the gene copy numbers (GCN) of the selected clones from MC-CBB-IA_cadA+mttA+mfsA$_{GAP}$ strain during the autotrophic production phase.

**Clone #**	**Final Titer (g L^{-1})**	**Specific productivity, q_p (mg g DCW^{-1} h^{-1})**	**Specific growth rate, μ (h^{-1})**	**Product yield (g g^{-1} DCW)**	**GCN of mfsA**	**GCN of cadA**
Single copy	1.670	4.369	0.008	0.527	1.0	1.0
Single copy	1.301	4.372	0.007	0.656	0.9	1.4
Single copy	1.960	5.350	0.009	0.597	1.4	1.1
MC_cadA#1	1.755	4.708	0.009	0.542	0.7	1.5
MC_cadA#2	1.726	4.363	0.008	0.519	2.0	0.6
MC_mfsA#1	2.223	6.312	0.008	0.756	6.0	1.0
MC_mfsA#2	2.117	5.169	0.008	0.619	3.9	0.9
MC_mfsA#3	1.417	3.797	0.008	0.491	4.4	1.0
MC_mfsA#4	1.940	5.387	0.007	0.730	5.0	1.0

MC_mfsA#5	1.845	5.147	0.007	0.702	5.1	1.0
MC_mfsA#6	1.622	4.819	0.007	0.647	4.2	1.0
MC_cadA#mfsA#1	2.191	5.71	0.008	0.653	3.0	2.1
MC_cadA#mfsA#2	2.147	5.63	0.009	0.649	1.5	1.6

Supplementary Table 2. Growth and itaconic acid production characteristics of the reverse engineered[1] (RE) strain with multicopy of *mfsA*. Data is calculated using the screening data at 25°C, with 10% CO_2. Genotypes of the strains are given in Table 2.

**Strain**	**Final Titer (g L^{-1})**	**Specific productivity, q_p (mg g DCW^{-1} h^{-1})**	**Specific growth rate, μ (h^{-1})**
RE	-	-	0.012
RE_cadA+mttA	0.826	1.100	0.010
RE_MC_IA_cadA+mttA+mfsA	1.365±0.324	1.784±0.344	0.010±0.000
MC-IA_cadA+mttA+$mfsA_{GAP}$	0.870±0.284	2.458±0.273	0.007±0.001

References

1 T. Gassler, M. Baumschabl, J. Sallaberger, M. Egermeier and D. Mattanovich, *Metab Eng*, 2022, 69, 112–121.
